# Retrospective Comparison of the Pericapsular Nerve Group Block Versus the Fascia Iliaca Block for Hip Fractures in the Emergency Department

**DOI:** 10.5811/westjem.53940

**Published:** 2026-09-24

**Authors:** Theresa Kim, Vahe Martikian, Soheil Saadat, Michael Hetzel, Matthew Whited, Ami Kurzweil

**Affiliations:** * Eisenhower Medical Center, Department of Emergency Medicine, Rancho Mirage, California; † University of California, Irvine, Department of Emergency Medicine, Orange, California

## Abstract

**Introduction:**

Hip fractures are a common injury among the elderly and a substantial cause of morbidity and mortality worldwide. Pain management is an essential component of care for patients with hip fractures, particularly for improving patient outcomes. Ultrasound-guided nerve blocks such as the fascia iliaca block (FIB) are evidence-based means of providing perioperative analgesia and reducing opioid use. Newer nerve blocks, such as the pericapsular nerve group (PENG) block, may serve as alternatives to the FIB. The objective of this study was to retrospectively compare pain reduction following the PENG block and FIB in patients with hip fractures treated in our emergency department. Secondary objectives included block-related complications such as local anesthetic systemic toxicity (LAST), greater hospital length of stay, and increase in mortality.

**Methods:**

The primary outcome measure was to investigate whether the PENG block is an effective alternative to FIB in achieving analgesia. Secondary objectives include data on rates of LAST, block complications, hospital length of stay, and mortality. We conducted a retrospective chart review on adult patients (≥18 years) with hip fractures who received either a FIB or PENG block between June 2021 and June 2023. Among the participants, pain scores (0–10 scale) were recorded before and after the block. Opioid use 24 hours postblock was also documented. We used generalized estimating equation modeling to compare pre-to-post block pain reduction between the FIB and PENG block groups.

**Results:**

A total of 88 participants met inclusion criteria (73 FIB, 15 PENG). The mean (SD) preblock pain scores were 6.5 (2.1) for FIB and 7.9 (1.6) for PENG, improving to 4.4 (2.0) and 4.3 (2.1) postblock, respectively. Generalized estimating equation analysis revealed a statistically significant greater reduction in pain for the PENG group compared to FIB (mean difference = 1.42; 95% CI, 0.21–2.63; *P* = .02). In this study, there were no cases of local anesthetic systemic toxicity or other block-related complications observed (0%; 95% CI, 0%–3.4%).

**Conclusion:**

Patients who received the PENG block achieved a greater reduction in pain scores compared to the FIB in this small retrospective cohort study. These findings suggest that the PENG block may be an effective alternative to FIB for analgesia in this setting. However, larger, prospective studies are needed to confirm these results. Given the study design and baseline differences in pain scores, these findings should be interpreted with caution.

## INTRODUCTION

Hip fractures continue to be a significant public health issue leading to considerable morbidity and mortality. The global incidence of hip fractures was estimated to be 14.2 million in 2019. The number of hip fractures is projected to increase 1.9-fold by 2050 compared to 2018.^1^

Pain management strategies in patients with hip fractures include ultrasound-guided regional nerve blocks. Two studies have shown that ultrasound-guided regional anesthesia (femoral nerve block and fascia iliaca block [FIB]) is more effective than parenteral or intramuscular morphine in a preoperative setting.^2^,^3^ A meta-analysis by Zaki et al demonstrated that the femoral nerve block and FIB provided more effective analgesic outcomes compared to parenteral opioids, with reduced need for rescue analgesia and fewer opioid-related adverse effects.^4^ Although FIB is considered the gold standard of care for analgesia in hip fracture patients, a newer regional nerve block—the pericapsular nerve group (PENG) block—provides promising results. The PENG block is injected directly into the pericapsular space between the psoas tendon and the pubic ramus (medial to the anterior inferior iliac spine), targeting the articular branches of the femoral, obturator, and accessory obturator nerves.^5^ The FIB is injected beneath the fascia iliaca, targeting the femoral, lateral femoral cutaneous, obturator, and genitofemoral nerves.^6^

Multiple studies have demonstrated that patients who received postoperative PENG blocks had lower pain scores (measured by the visual analog scale), reduced total opioid consumption, and received higher postoperative analgesia satisfaction scores compared to those who received the FIB.^7^,^8^ Sahoo et al compared analgesic effectiveness and adverse effects between PENG blocks and intravenous opioids in the emergency department (ED), demonstrating further evidence of improved analgesia, based on numerical rating scale pain scores, and fewer adverse effects with PENG blocks.^9^ The PENG block is becoming a popular alternative to the FIB for pain control; however, further studies are needed to compare the PENG block with other analgesic methods in the ED setting.

The primary objective was to evaluate retrospectively whether PENG blocks were an effective alternative to the FIB for analgesia in patients who presented to the ED with hip fractures. Secondary objectives included data on rates of local anesthetic systemic toxicity (LAST), block complications, hospital length of stay (LOS), and mortality.

## METHODS

### Study Design

This study is a retrospective chart review on participants who received a PENG block or FIB during their ED hospitalization for hip fracture. Data were extracted using the SlicerDicer feature in the electronic health records (EHR) software (Epic Systems Corporation), with a date range from June 2021–June 2023. Institutional review board approval was obtained on September 27, 2023. We collected data on pain level and opioid usage before and after the procedure. All data were collected from patient visits to the ED of Eisenhower Medical Center, a community hospital with an annual volume of approximately 100,000. Evaluation of these patients demonstrated a distribution of six postgraduate year (PGY) 1, five PGY-2, six PGY-3 emergency medicine (EM) residents, and four EM attending clinicians. The chart review was conducted by an ultrasound fellowship-trained emergency physician.

**Population Health Research Capsule**

What do we already know about this issue?

*Ultrasound-guided fascia iliac blocks (FIB) reduce pain in hip fracture patients. The newer pericapsular nerve group (PENG) block has limited studies on efficacy*.

What was the research question?

*Do PENG blocks provide greater analgesia than fascia iliaca blocks for emergency department (ED) hip fracture patients?*

What was the major finding of the study?

*The PENG block reduced pain 1.42 points more than FIB (95% CI, 0.21–2.63; P = .02)*.

How does this improve population health?

*The PENG block may improve pain control for hip fracture patients in the ED and can be another option to FIB*.

Inclusion criteria were patients 18 years of age or older who presented with a hip fracture and received a FIB/PENG block in the ED. Exclusion criteria were patients who were intubated or unable to verbalize pain score because of underlying pathology, those with no documented block type, and those whose nerve blocks were performed outside the ED or by a non-EM physician. Patients were also excluded if they lacked recorded preblock or postblock pain scores, block times, or local anesthetic dosages.

We collected data on patient demographics, type of injury, type of nerve block (PENG or FIB), pain scores, local anesthetic dosages, and 24-hour opioid use during hospitalization.

### Data Collection

The methodological recommendations described by Worster et al were considered during this retrospective chart review.^10^ We used an Excel spreadsheet to record data. The abstractor was trained on data collection and chart review through the EHR system. Inclusion and exclusion criteria were defined. Charts were de-identified using the last four digits of the medical record number. We recorded the block type, anesthetic dosage, age, sex, medical history, diagnosis, morphine milligram equivalents (MME) in 24 hours, LOS, and complications. However, given that this retrospective review was completed by a single abstractor, interrater reliability testing was not performed. The abstractor was not blinded to the study objectives.[Fig f1-wjem-27-1485]

### Statistical Analysis

Pain was recorded on a 0–10 numeric rating scale repeatedly preblock and postblock. Because measurement frequency and timing varied across participants, we calculated an average pain score for each patient before and after the nerve block. We compared changes in pain intensity following the block (calculated as the average pain score postblock minus the average pain preblock) between the FIB and PENG groups; a generalized estimating equation was used to account for the potential correlation between preblock and postblock pain scores. The study group (FIB vs PENG), time period (preblock vs postblock), and their interaction were included as fixed factors in the model. Model-based marginal means by group and period, as well as within-group changes, were derived from the fitted model. We specified a Gaussian family with an identity link and an exchangeable correlation structure to account for the clustering of preblock and postblock observations within each patient. No imputation was performed. Analysis was performed with SPSS Statistics for Windows, version 31.0 (IBM Corp). All reported *P* values are two-sided.

## RESULTS

### Study Sample

We selected 127 patients for review, identifying 110 FIB and 17 PENG blocks. Of the 110 FIB patients, 37 were excluded for the following reasons: one hip dislocation; one with no ropivacaine administration time recorded; one done by anesthesia; one with no pain score; 9 with no nursing documentation of the block; and 24 with no pain scores recorded preblock or postblock. Two PENG block candidates were excluded due to lack of recorded pain scores preblock or postblock. Included in the final analysis were 73 patients in the FIB group and 15 in the PENG group. The mean pain score before the nerve block was 6.5 in the FIB group and 7.9 in the PENG group, which decreased to 4.4 and 4.3, respectively (Figure 2).

### Generalized Estimating Equation Analysis

A total of 88 patients were included for the generalized estimating equation analysis. The interaction between the study group and preblock/postblock was statistically significant (Wald χ^2^ = 5.28; df = 1; *P* = .02), indicating a greater pre-to-post reduction in pain in the PENG group than in the FIB group (Table 1).

### Safety Outcomes

There were no cases of local anesthetic systemic toxicity or other block-related complications in this study of 88 patients. The observed complication rate was 0% (95% CI, 0% – 3.4%).

As shown in Table 2, pain in the PENG group improved by 1.42 points more than the FIB group (95% CI, 0.21–2.63; *P* = .02).

## DISCUSSION

This retrospective comparative study between the PENG block and FIB in patients who sustained hip fractures demonstrated improved pain scores in the PENG group compared to the FIB group. Mean pain scores before the nerve block were 6.5 in the FIB group and 7.9 in the PENG group with improvement to 4.4 and 4.3, respectively, with statistical significance. This suggests that the PENG block was more effective than the FIB in pain control. Although the PENG group in our study was relatively small (*n* = 15), supporting evidence from previous prospective studies showed equivalence in anesthesia between PENG and FIB. One study involving 40 patients in each group demonstrated similar analgesic efficacy between FIB and PENG.^11^ Of the 88 patients, none experienced block-related complications, which include LAST, prolonged hospital LOS, or increased mortality.

Our data suggest an association between the PENG block and improved analgesia in preoperative patients in a community ED. Prior studies have also found that the PENG block provides greater postoperative pain relief with less quadriceps muscle weakness in patients with intertrochanteric femoral fractures.^7^,^12^ These motor-sparing properties can lead to improved postoperative recovery times and earlier mobilization. However, the clinical significance of these findings is unclear because other studies have found no significant differences in overall recovery between the FIB and the PENG block, despite the preserved quadriceps function.^13^

## LIMITATIONS

One key limitation of our study is the nature of a retrospective chart review. Pain score assessment times were inconsistent; therefore, the precision or quality of data is not as robust compared to a prospective study. Several recommendations for retrospective chart review methodology described by Worster et al were followed. However, not all quality measures were possible. For instance, interrater reliability was not assessed because chart review was performed by one reviewer. Additionally, the abstractor was not blinded to the hypothesis, and no independent validation of data abstraction was performed. These points are important to consider because of measurement bias. Another key limitation is our small sample size, with 73 patients in the FIB group compared to 15 in the PENG group. The fascia iliaca block is more commonly performed by emergency clinicians. Additionally, preblock pain scores were higher in the PENG block group (7.9 vs 6.5), which raises the possibility of regression to the mean effects.

We analyzed the results with generalized estimating equation modeling. However, no additional adjustments for fracture type, time to block, anesthetic dose variability, clinician experience, or opioid use prior to block were made for baseline differences between the groups. Furthermore, baseline imbalance in preblock pain scores could have introduced the possibility of selection bias. With regard to missing data, we excluded outliers where no pain scores were recorded preblock or postblock.

Four data points demonstrated high MME postblock in patients with a history of chronic myeloid leukemia, rheumatoid arthritis, breast cancer, alcohol or opioid use disorder, and chronic pain. Three patients had more MME prior to the nerve block; however, these individuals had a delay in nerve block administration of at least three hours after presenting to the ED. Seven patients had a baseline score of zero preblock but reported mean pain score postblock.

A total of 21 data points demonstrated worsening pain scores postblock. It is possible that the training level of the administering physician could have affected the administration of both nerve blocks. The operators included six PGY-1, five PGY-2, six PGY-3 EM residents, and four EM attending clinicians. We were unable to control for the prior ultrasound-guided procedural experience of the physicians performing the nerve blocks.

Finally, although we did not observe block-related complications, our study was not sufficiently powered to assess safety of the procedure.

## CONCLUSION

Patients who received the PENG block achieved a greater reduction in pain scores compared to the FIB. The PENG block was associated with greater reduction in mean pain scores. Future studies should take into consideration the training level of emergency clinicians, assess for variables including multiple comorbidities and chronic pain, and gather data with complete chart documentation. A future randomized controlled trial could provide a blinded comparison of the two nerve blocks alongside the evaluation of total time required for nerve block setup and completion. Larger sample sizes are needed to determine whether the PENG block provides greater pain contol, functional recovery, and long-term outcomes.

## Figures and Tables

**Figure 1 f1-wjem-27-1485:**
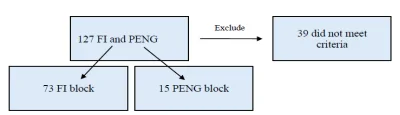
Flow diagram of participant inclusion and exclusion in the pericapsular nerve group versus fascia iliaca block study. *FI*, fascia iliaca; *PENG*, pericapsular nerve group.

**Figure 2:**
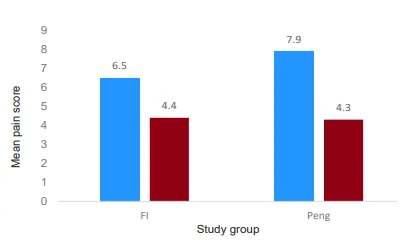
Average pain score before and after nerve block, by study group in the pericapsular nerve group versus fascia iliaca block study. The graph shows pain scores as observed, without any adjustment. Adjusted pain scores are reported in Table 2.

**Table 1:** Generalized estimating equation analysis.

Source	Type III		

	Wald χ^2^	df	*P* value
(Intercept)	623.140	1	< .001
Study group	3.278	1	.070
Pre/post Injection	81.114	1	< .001
Study group * Pre/post Injection	5.280	1	.02

*P* value for the interaction between study group and preblock/postblock indicates a difference in pain score change after the block between the study groups.

We used the Wald χ^2^ statistic to compute *P* value.

**Table 2:** Estimated marginal means before and after nerve block, by study group.

Study Group	Pre/Postblock	Mean	SE	95% Wald CI (Lower)	95% Wald CI (Upper)
PENG	Post	4.40	0.47	3.48	5.32
PENG	Pre	7.89	0.46	6.98	8.80
FIB	Post	4.28	0.24	3.80	4.76
FIB	Pre	6.35	0.34	5.68	7.01

*FIB*, fascia iliaca block; *PENG*, pericapsular nerve group; *SE*, standard error.

## References

[1] Sing CW, Lin T-C, Bartholomew S (2023). Global epidemiology of hip fractures: secular trends in incidence rate, post-fracture treatment, and all-cause mortality. J Bone Miner Res.

[2] Beaudoin FL, Haran JP, Liebmann O (2013). A comparison of ultrasound-guided three-in-one femoral nerve block versus parenteral opioids alone for analgesia in emergency department patients with hip fractures: a randomized controlled trial. Acad Emerg Med.

[3] Foss NB, Kristensen BB, Bundgaard M (2007). Fascia iliaca compartment blockade for acute pain control in hip fracture patients: a randomized, placebo-controlled trial. Anesthesiology.

[4] Zaki HA, Iftikhar H, Shallik N (2022). An integrative comparative study between ultrasound-guided regional anesthesia versus parenteral opioids alone for analgesia in emergency department patients with hip fractures: a systematic review and meta-analysis. Heliyon.

[5] Ben Aziz M, Mukhdomi J (2024). Pericapsular nerve group block. StatPearls.

[6] Ruzbarsky JJ, Gausden EB, Goldwyn EM (2018). The fascia iliaca block as the primary intraoperative anesthesia for hip fracture surgery: a preliminary study. HSS J.

[7] Kong M, Tang Y, Tong F (2022). The analgesic efficacy of pericapsular nerve group block in patients with intertrochanteric femur fracture: a randomized controlled trial. PLoS One.

[8] Mosaffa F, Taheri M, Manafi Rasi A (2022). Comparison of pericapsular nerve group (PENG) block with fascia iliaca compartment block (FICB) for pain control in hip fractures: a double-blind prospective randomized controlled clinical trial. Orthop Traumatol Surg Res.

[9] Sahoo S, Sahoo NK, Hansda U (2024). Ultrasound-guided pericapsular nerve block compared with IV opioids in hip injuries: a randomized controlled trial. Am J Emerg Med.

[10] Worster A, Bledsoe RD, Cleve P (2005). Reassessing the methods of medical record review studies in emergency medicine research. Ann Emerg Med.

[11] Koh WU, Kim H, Kim YJ (2024). Comparison of analgesic effect of pericapsular nerve group block and supra-inguinal fascia iliaca compartment block on dynamic pain in patients with hip fractures: a randomized controlled trial. Reg Anesth Pain Med.

[12] Lee TY, Chung CJ, Park SY (2024). Comparing the pericapsular nerve group block and the lumbar plexus block for hip fracture surgery: a single-center randomized double-blinded study. J Clin Med.

[13] Lin DY, Morrison C, Brown B (2021). Pericapsular nerve group (PENG) block provides improved short-term analgesia compared with the femoral nerve block in hip fracture surgery: a single-center double-blinded randomized comparative trial. Reg Anesth Pain Med.

